# Glial Cell Contributions to Auditory Brainstem Development

**DOI:** 10.3389/fncir.2016.00083

**Published:** 2016-10-21

**Authors:** Karina S. Cramer, Edwin W Rubel

**Affiliations:** ^1^Department of Neurobiology and Behavior, University of California, IrvineIrvine, CA, USA; ^2^Virginia Merrill Bloedel Hearing Research Center, University of WashingtonSeattle, WA, USA

**Keywords:** nucleus laminaris, nucleus magnocellularis, medial nucleus of the trapezoid body, astrocyte, oligodendrocyte, delay line, microglia, calyx of Held

## Abstract

Glial cells, previously thought to have generally supporting roles in the central nervous system, are emerging as essential contributors to multiple aspects of neuronal circuit function and development. This review focuses on the contributions of glial cells to the development of auditory pathways in the brainstem. These pathways display specialized synapses and an unusually high degree of precision in circuitry that enables sound source localization. The development of these pathways thus requires highly coordinated molecular and cellular mechanisms. Several classes of glial cells, including astrocytes, oligodendrocytes and microglia, have now been explored in these circuits in both avian and mammalian brainstems. Distinct populations of astrocytes are found over the course of auditory brainstem maturation. Early appearing astrocytes are associated with spatial compartments in the avian auditory brainstem. Factors from late appearing astrocytes promote synaptogenesis and dendritic maturation, and astrocytes remain integral parts of specialized auditory synapses. Oligodendrocytes play a unique role in both birds and mammals in highly regulated myelination essential for proper timing to decipher interaural cues. Microglia arise early in brainstem development and may contribute to maturation of auditory pathways. Together these studies demonstrate the importance of non-neuronal cells in the assembly of specialized auditory brainstem circuits.

## Introduction

Our ability to localize sound sources relies in large part on specialized circuitry in the auditory brainstem. Unlike other sensory modalities, such as the visual and somatosensory systems, in which spatial information is encoded in the sensory epithelium, the cochlea is unique in that it instead contains an orderly representation of frequency selectivity. This frequency map is conveyed through spiral ganglion cells to the brainstem, and interaural time and intensity differences are computed through specialized circuitry in the superior olivary complex. The development of these specialized neural pathways requires coordination of multiple molecular and cellular mechanisms. Several classes of axon guidance molecules have been identified that contribute to the formation of synaptic connections with precisely selected targets. Emerging evidence has shown that several classes of glial cells, including astrocytes, oligodendrocytes and microglia, play important roles in nervous system development (Stevens, 2008; Allen, 2013; Clarke and Barres, 2013; Edmonson et al., 2016). Here we review recent studies that have begun to demonstrate the contributions of these non-neuronal cells to multiple aspects of auditory brainstem circuit assembly.

## Chick Auditory Brainstem Pathways

In vertebrates, sound stimuli are transduced in the ear, where auditory hair cells in the cochlea are spatially ordered according to their preferred stimulus frequency. Hair cells synapse onto peripheral processes of cochlear ganglion cells, whose central processes enter the brainstem through the eighth cranial nerve (nVIII) and contact the cochlear nuclei, preserving tonotopy. Birds and mammals both use interaural time differences (ITDs) and interaural level differences (ILDs) to varying degrees to infer the locations of sound sources in space.

### ITD Circuitry in the Chick

**Figure 1:**
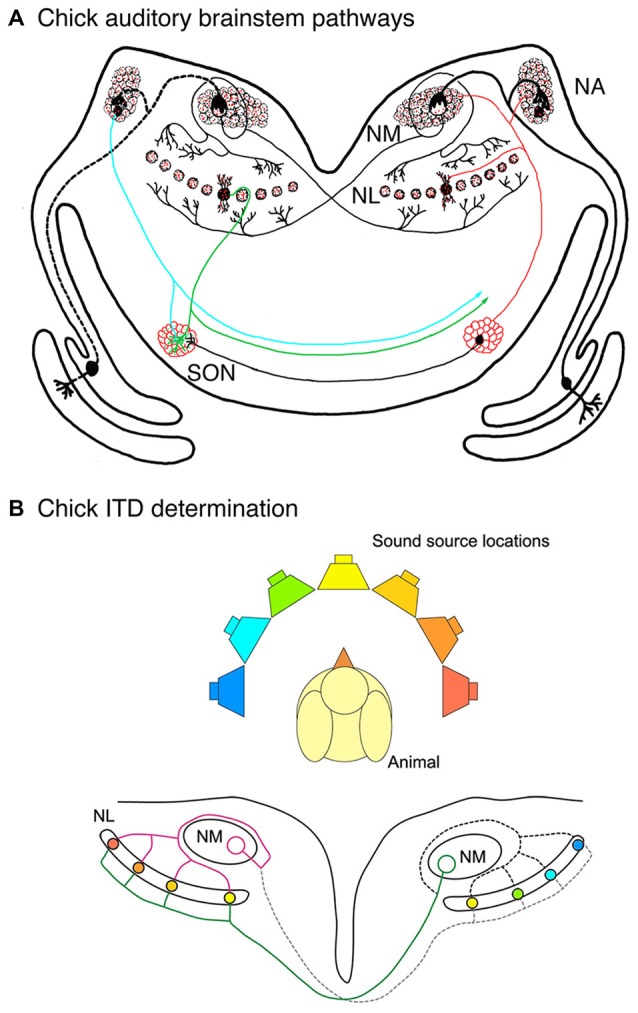
**Auditory brainstem pathways used in sound localization in the chick. (A)** Auditory nerve axons innervate ipsilateral nucleus magnocellularis (NM) neurons, which in turn innervate nucleus laminaris (NL) bilaterally. Ipsilateral and contralateral NM axon branches make contact with dorsal and ventral NL dendrites, respectively. Ventral NM axon branches display delay lines and dorsal axon branches course around NM before making their contacts. Inhibitory input is largely provided by the superior olivary nucleus (SON), shown on the left side. **(B)** Delay lines in NM axons and coincidence detection in NL neurons are key factors in determination of interaural time difference (ITD) determination. Coincidence detection in NL neurons varies along the mediolateral axis depending on the location of the sound source relative to the animal. NL neurons with coincident input and the corresponding locations are shown here in matching colors.

The chick pathway, shown in Figure 1, demonstrates the specialized spatial arrangement of auditory brainstem connectivity that allows for computation of ITDs. The key circuit element is the projection from nucleus magnocellularis (NM; Figure 1A), which receives ipsilateral nVIII input, to nucleus laminaris (NL), which contains a sheet of bitufted neurons with dorsal and ventral dendrites (Smith and Rubel, 1979; Jhaveri and Morest, 1982). NM neurons project through a bifurcated axon to NL on both sides of the brainstem. Ipsilateral NM axon branches synapse onto the dorsal NL dendrites, whereas contralateral NM axon branches synapse onto ventral NL dendrites (Parks and Rubel, 1975; Hackett et al., 1982; Young and Rubel, 1983). The contralateral NM axon branches introduce delay lines so that conduction time is longer to reach more lateral NL neurons than the more medial neurons (Overholt et al., 1992). Highly coincident input from the two NMs is needed for NL neuronal firing. The arrangement of the NM-NL pathway results in a correlation between the sound source and the location of NL neurons receiving coincident input (Figure 1B). Sounds presented close to one ear will result in coincident input and activation of lateral NL neurons contralateral to that ear, while sounds presented directly ahead will activate medial NL neurons on both sides of the brain (Carr and Konishi, 1990; Hyson, 2005).

Several additional organizing features enhance the function of the NM-NL pathway in ITD computation (Ohmori, 2014). Coincidence detection in NL neurons is improved with inhibitory input, which arises mainly from axons of the superior olivary nucleus (SON) in the ventral region of the auditory brainstem (Lachica et al., 1994; Yang et al., 1999; Burger et al., 2011). The spatial arrangement of best ILDs is orthogonal to the frequency axis, in which high frequencies are represented in rostromedial NL and low frequencies are represented in caudolateral NL. While NL dendrites generally show symmetry for the dorsal and ventral sides, a steep gradient of dendritic arbor size emerges late in embryonic development (Smith and Rubel, 1979; Smith, 1981). Mature NL neurons have fewer and longer dendrites in the low frequency region and numerous short dendrites in the high frequency region. This specialization is thought to optimize coincidence detection for each frequency (Agmon-Snir et al., 1998).

### Astrocytes in the Developing Chick Auditory Brainstem

Glial cells are found throughout the auditory brainstem during the formation and maturation of the NM-NL pathway and are well positioned to contribute to most of the spatially ordered features of the auditory circuitry described above. Astrocytes are seen in the brainstem when NM and NL are still part of a common cell group known as the auditory anlage, at about embryonic day (E) 8. Glial fibers express the intermediate filament vimentin, a marker for radial glia. At E10, when NM-NL connections form, vimentin expression is limited to the dorsal region of NL (Korn and Cramer, 2008), coinciding with the ipsilateral NM input. This intriguing pattern suggests a potential role for glial fibers in appropriate segregation of ipsilateral and contralateral NM axon branches to dorsal and ventral regions of NL.

#### NM Axons and NL Glia

**Figure 2:**
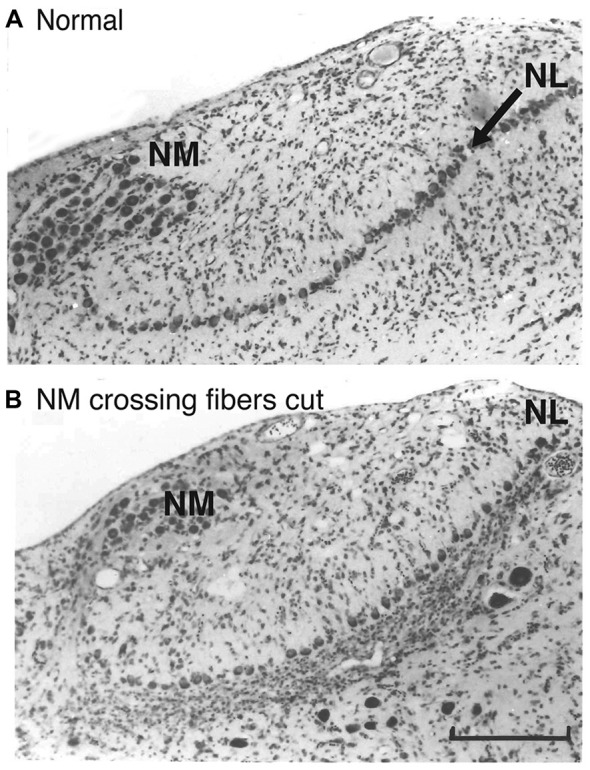
**The glial margin surrounding NL is disrupted by deafferentation. (A)** Normal Nissl stained coronal section illustrating NM and the monolayer of cell bodies in NL (arrow). A relatively cell body-free neuropil zone is bordered by densely packed, small glial cells. **(B)** Eight days after a tract cut at the midline, a massive movement of glial cells into the denervated ventral region was observed. Adapted from Rubel et al. (1981). Scale bar, 50 μm, applies to both panels.

Further suggestion of a role for glia in NL axon guidance is seen in the glial cell bodies around NL. NM and NL separate from each other in the auditory anlage at about E10, and NL forms a monolayer surrounded by neuropil composed of dendrites, axonal terminal processes and glial processes that is largely devoid of cell bodies. This neuropil region is surrounded by a dense margin of small glial cells (Rubel et al., 1976) that remain outside the neuropil until later embryonic ages (Figure 2A). These glial cells were shown to express the axon guidance molecule ephrin-B2 (Person et al., 2004). Moreover, the expression of ephrin-B2 displays a concentration gradient corresponding with the tonotopic axis of NL, alongside a similar ephrin-B2 expression gradient in the adjacent neurons of NL. The role of NL glial cells in axon guidance has not been critically tested. However, when Eph family proteins were experimentally inhibited during development, we found that mistargeting of NM axons was accompanied by disruption of NL morphogenesis, suggesting that the structural integrity of NL and its glial margin are linked to axon growth (Cramer et al., 2004; Allen-Sharpley and Cramer, 2012). This link is further supported by experiments in which the crossing NM axon branches were severed (Figure 2B), resulting in deafferentation of the ventral NL dendrites (Rubel et al., 1981). The resulting rapid atrophy of these dendrites was accompanied by a substantial invasion of glial cells into the ventral cell-free neuropil region. NL glia thus define a boundary around the nuclei, and the integrity of this boundary is dependent on innervation from NM.

#### Morphology of NL Dendrites

The topographic gradient of ephrin-B2 in glia surrounding NL suggests a potential role for these cells not only in axon targeting, but perhaps also in other features that are graded along the frequency axis. We explored one such feature, the gradient of NL dendritic arbor morphology that emerges in late embryonic development. We reasoned that the relatively late appearance of glial fibrillary acidic protein (GFAP)-positive astrocytes coincides with this process, and we tested the effect of GFAP-positive astrocyte conditioned medium (ACM) on dendritic maturation in an organotypic auditory brainstem slice culture that permitted repeated imaging of individual labeled dendrites (Korn et al., 2011). We prepared slices from E13 embryos, when few GFAP-positive astrocytes are seen, and exposed them to ACM from astrocytes purified from more mature brainstems. We found that in slices cultured with ACM, dendrites in NL exhibited a loss of primary dendrites, with a greater effect in caudolateral (low frequency) regions, whereas control cultures did not show a significant change over the same period. The results suggest that this aspect of dendritic maturation is regulated by secreted signals from astrocytes. The graded nature of the effect, obtained with bath application of ACM, indicates that NL cells vary in their responsiveness to astrocytic factors. Orthogonal to this axis, the dorsoventrally oriented glial fibers seen in mature NL have been postulated to contribute to the bidirectional orientation of NL dendrites (Kalman et al., 1998). Glial influences may thus contribute to several aspects of dendrite arbor morphology.

#### Inhibitory Synapses in NL

In addition to these studies of dendritic regulation, we used our organotypic preparation to study the effects of ACM on synaptogenesis (Korn et al., 2012). We found that inhibitory synapses begin forming in NL later than excitatory synapses, and they become more abundant at ages when GFAP-positive astrocytes are present. Inhibitory inputs from SON were quantified with counts of puncta immunolabeled for the vesicular GABA transporter (VGAT). We found that ACM resulted in a significantly enhanced rate of inhibitory synaptogenesis compared to control cultures. Notably, the treated cultures added inhibitory synapses at a time course similar to that seen *in vivo*, suggesting that astrocyte secreted factors may mediate progression of inhibitory synapse formation. The identity of these factors remains to be determined.

### Myelination and its Regulation in Sound Localization Pathways

Conduction timing in the NM-NL pathway is a critical factor in ITD detection. It is especially complicated by the fact that coincidence is needed from two distinct inputs, one from the nearby ipsilateral NM and the other from the distant contralateral NM. Early studies showed that the ipsilateral NM axon branch loops around NM before arriving in NL (Young and Rubel, 1986), thereby increasing the conduction time to make it more similar to the contralateral branch. However, several additional factors are needed to adjust this timing. Recently, precise measurements of axon length incorporating 3-dimensional reconstructions showed that the contralateral NM axon branches are roughly twice the length of the ipsilateral branch in the chick (Seidl et al., 2010; Seidl, 2014). Remarkably, the two NM axon branches differed in diameter and in the spacing between nodes of Ranvier, resulting in differences in conduction velocity to optimize temporal integration (Seidl et al., 2014). The regulation of axon thickness and internodal distance demonstrates a critical role for myelinating oligodendrocytes in the establishment of the functional pathway.

While the mechanisms that regulate spacing between nodes of Ranvier in this pathway are not known, some of the factors that guide myelination during development are beginning to be understood. Myelination is regulated by activity-dependent communication between neurons and oligodendrocytes, both during initial development and in adult life (Barres and Raff, 1993; Lin and Bergles, 2004; Fields, 2015; Hines et al., 2015). Myelination of NM axons in the chick embryo (Korn and Cramer, 2008) and particularly in the barn owl (Cheng and Carr, 2007) occurs relatively late in development, after the onset of neuronal activity. The link between neuronal activity and myelination may result directly from the non-synaptic release of neurotransmitters that bind to receptors on oligodendrocytes. Activity may also act by increasing expression of molecules that have necessary roles in myelination, such as brain derived neurotrophic factor (BDNF) and the cell adhesion molecule L1CAM (Barbin et al., 2004; Wong et al., 2013; Purger et al., 2015). The remarkable and important contribution by the studies on the chick and mammalian studies (see below) on the brainstem ITD pathways is that the ipsilateral and contralateral processes of *the same axon* are independently regulated. These findings indicate that the molecular cues must act locally on individual branches rather than through the cell bodies of the glial cells.

## Mammalian Auditory Brainstem Pathways

As in birds, the mammalian auditory brainstem displays precise innervation and specialized synaptic structures that facilitate sound source localization. Glial cells contribute to the maturation and function of these pathways.

### Myelination and its Regulation in Mammalian Pathways

**Figure 3:**
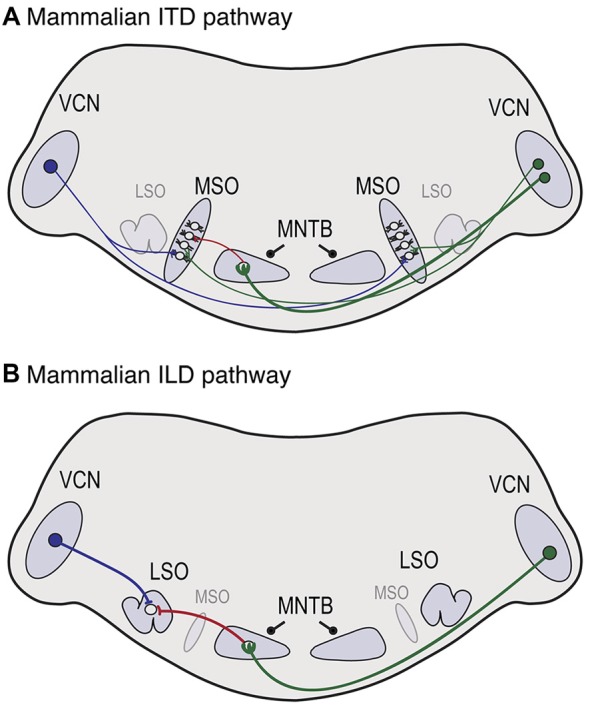
**Neuroanatomy of mammalian auditory brainstem pathways used in sound localization. (A)** In the ITD pathway, neurons in the ventral cochlear nucleus (VCN) project axons that branch and provide excitatory input to the medial superior olive (MSO) on both sides of the brain, with ipsilateral and contralateral inputs to lateral and medial MSO dendrites, respectively. Globular bushy cells (GBCs) in VCN synapse with neurons in the medial nucleus of the trapezoid body (MNTB) on the contralateral side, where they terminate in the calyx of Held, shown on the left side. MNTB provides one source of inhibitory input to MSO neurons. **(B)** In the interaural level difference (ILD) pathway, lateral superior olive (LSO) neurons receive excitatory input from spherical bushy cells (SBCs) in ipsilateral VCN and inhibitory input from ipsilateral MNTB. Because sounds are louder near the sound source, the LSO on the side of the sound source receives more excitation and less inhibition, while the opposite holds in the LSO more distant from the sound source.

Action potential timing is also important in mammalian sound localization pathways, shown in Figure 3. In the mammalian ITD pathway (Figure 3A), spherical bushy cells (SBCs) in the anterior ventral cochlear nucleus (VCN) send axons that branch and make excitatory contacts with neurons in the medial superior olive (MSO) on both sides of the brain. A recent study in gerbils demonstrated that, like NM axons, contralateral SBC axon branches projecting to MSO have thicker axon diameters and longer internode distances (Seidl and Rubel, 2016). The difference in axon thickness was first observed at postnatal day (P) 20. In contrast, the difference in internode distance was already evident at P10, which is prior to hearing onset (Woolf and Ryan, 1984). If activity-dependent regulation is important for this aspect of myelination, it may rely on spontaneous neuronal activity, which is generated early in development (Tritsch et al., 2010; Wang and Bergles, 2015). It is also important to consider later maturation because the relative timing between the two ears will be altered by changes in head size. This aspect has not been studied to date and it will likely involve a change in the distribution of nodes that depends on activity.

In the ILD pathway (Figure 3B), SBCs project to the lateral superior olive (LSO) on the ipsilateral side. A major component of both pathways includes inhibition from the ipsilateral medial nucleus of the trapezoid body (MNTB), a sign reversing relay nucleus that receives excitatory input from globular bushy cells (GBCs) in contralateral VCN (Kuwabara and Zook, 1991; Grothe and Sanes, 1993, 1994; Pecka et al., 2008). The long axon of the GBC neuron terminates in MNTB in a large specialized synaptic ending known as the calyx of Held (Held, 1893; Kuwabara and Zook, 1991; Kuwabara et al., 1991). LSO neurons receive excitatory input from the ipsilateral side, and inhibitory input originating from the contralateral side, and the balance of excitation and inhibition is used to determine ILDs (Tollin, 2003). Although the disynaptic inhibitory input from MNTB is longer and subject to synaptic delay, it arrives at the same time as the monosynaptic excitatory inputs, and even early in MSO (Roberts et al., 2013, 2014). A recent study (Ford et al., 2015) demonstrated that GBCs have thicker axon diameters and increased internodal distances compared to SBCs, accounting for this disparity. Additionally, they found systematic differences in these properties that varied with GBC projections along the mediolateral (high frequency to low frequency) axis of MNTB and showed that these variations provide optimal conduction times for these tonotopic locations. Thus in both ITD and ILD pathways, axon thickness and myelination are adjusted to generate precise timing.

### Ontogeny of Glial Cells in the Auditory Brainstem

Because of the wide range of glial functions involved in neuronal development, we characterized glial cell populations at key stages of auditory brainstem development. Oligodendrocytes and their precursors, identified with expression of oligodendrocyte transcription factor 2 (OLIG2), are seen in VCN and MNTB during the first postnatal week in mice (Dinh et al., 2014; Kolson et al., 2016). In rats, myelin basic protein is expressed in MNTB beginning at about P9 (Saliu et al., 2014), consistent with previous reports that myelination begins at about that age (Leão et al., 2005). In gerbils, myelin associated proteins are expressed in LSO during the first postnatal week, after which they display oriented patterns consistent with a role in guiding dendritic orientation of LSO neurons (Hafidi et al., 1996).

Along with oligodendrocytes, subpopulations of astrocytes are found in auditory nuclei during the early postnatal period in mice. As early as P0 we identified astrocytes in VCN and MNTB that were immunolabeled for aldehyde dehydrogenase 1 family member L1 (ALDH1L1), a specific marker that broadly labels astrocytes (Cahoy et al., 2008; Dinh et al., 2014; Kolson et al., 2016) and these cells increased in number during brainstem maturation. In contrast, we did not observe expression of the calcium binding protein, S100ß, or the intermediate filament, GFAP, two other astrocyte markers, in either nucleus at P0. S100ß-positive astrocytes were abundant in both VCN and MNTB by P6 (Dinh et al., 2014). In rats, S100ß-positive astrocytes were found in MNTB during the first postnatal week, where expression of this protein appears to be temporally correlated with the appearance of a group of non-neuronal cells that proliferate in the nucleus at this age (Saliu et al., 2014). As we found in chick embryos, GFAP expression was seen at later ages, with expression in VCN and MNTB first evident at about P14–23 in mice (Dinh et al., 2014). The GFAP expression revealed astrocytic fibers that coursed throughout the nucleus. In contrast, ALDH1L1 immunolabel at these ages appeared to coalesce densely around MNTB principal neurons. Vital labeling of astrocytes using sulforhodamine showed that they are present in LSO as early as P3, arranged in dense clusters of cells that express both GABA and glycine transporters and sequester inhibitory neurotransmitters (Stephan and Friauf, 2014). Together these studies reveal multiple populations of astrocytes throughout development. Their varied appearance and unique expression patterns suggest a range of functions in these developing nuclei.

A distinct population of glial cells, the microglia, also populate the auditory brainstem nuclei at early ages (Dinh et al., 2014). These cells, visualized with an antibody that recognizes the ionized calcium binding adaptor molecule 1 (IBA1), are found in small numbers in VCN at P0 and in MNTB at P6. Microglia in VCN and MNTB increase in number and in the abundance of ramified processes until they peak at about P14 and subsequently taper off in number. Both astrocytes and microglia are thus present at the time of hearing onset and both cell types are found in close apposition to the developing calyx of Held.

### Glial Cells and the Calyx of Held

Glial cells form close associations with the calyx of Held. In mature animals, the calyx has a fenestrated morphology, with astrocytic processes labeled in openings in the calyx (Ford et al., 2009). Along with S100ß, ALDH1L1 and GFAP, astrocytes associated with the mature calyx also express glutamate transporters that result in sequestration of glutamate and release of glutamine, which would improve temporal precision at the calyx. (Ford et al., 2009; Uwechue et al., 2012; Dinh et al., 2014). Both astrocytes and microglia are seen in close apposition with the developing calyx of Held (Dinh et al., 2014), and ultrastructural studies have shown that long glial processes cover MNTB neurons at early postnatal ages, in some cases interposed between nascent calyxes and the postsynaptic neuronal surface (Holcomb et al., 2013). Similar associations with astrocytic processes are seen in the large synaptic endings of auditory nerve inputs to VCN known as the endbulbs of Held. These astrocytic processes are remarkably dynamic. Studies in the chick cochlear nucleus showed that the astrocytic process grow and retract dramatically within hours after changes in eighth nerve activity (Canady and Rubel, 1992; Rubel and MacDonald, 1992). An ultrastructural analysis further showed that when activity is dramatically reduced glial process rapidly intercalate between the axonal contacts and the postsynaptic neurons (Canady et al., 1994).

While GBC projections to MNTB are usually limited to the contralateral side, early unilateral removal of the cochlea leads to loss of VCN neurons on the lesioned side (Trune, 1982; Hashisaki and Rubel, 1989; Mostafapour et al., 2000) and subsequent branching of intact VCN axons that leads to induction of calyceal terminations in their ipsilateral MNTB (Kitzes et al., 1995; Russell and Moore, 1995; Hsieh et al., 2007). Both astrocytes and microglia show a similar proximity to the developing induced calyces (Dinh et al., 2014). The rapid appearance of the ipsilateral calyx and its association with glial cells precedes a measurable glial response to the injury. Glial cells in MNTB thus appear to have a distinct role in synaptic development, and this role can be activated for ipsilateral inputs when the normally occurring contralateral inputs are lost.

## Concluding Remarks

While specialized auditory pathways engage glial cells for their mature function, glial cells also contribute significantly to the assembly of these pathways. Recent studies have shown roles for glial cells in synaptogenesis and dendrite maturation. Additionally, developmental regulation of myelination and internode distance are critical factors in establishing conduction times required for temporal processing in sound localization pathways of birds and mammals. While much remains to be discovered on the mechanisms of neuron-glial communication in these developmental processes, progress has been made determining the effects of this communication at numerous points of contact. Glial cells are integral components of functioning auditory brainstem pathways. These recent studies suggest that subpopulations of glia contribute to brainstem development and to their integration into these circuit elements.

## Author Contributions

KSC drafted and edited the manuscript and prepared figures. EWR contributed to revision of the manuscript and preparation of figures.

## Conflict of Interest Statement

The authors declare that the research was conducted in the absence of any commercial or financial relationships that could be construed as a potential conflict of interest.
